# Calcific panniculitis and nasopharyngeal cancer-associated adult-onset dermatomyositis: a case report and literature review

**DOI:** 10.1186/s40064-015-0994-7

**Published:** 2015-04-30

**Authors:** Manasmon Chairatchaneeboon, Kanokvalai Kulthanan, Araya Manapajon

**Affiliations:** Department of Dermatology, Faculty of Medicine Siriraj Hospital, Mahidol University, 2 Wanglang Road, 10700 Bangkok, Thailand

**Keywords:** Adult, Calcinosis, Dermatomyositis, Nasopharyngeal neoplasm, Panniculitis

## Abstract

Panniculitis is an uncommon cutaneous manifestation in dermatomyositis. It not only occurs in idiopathic dermatomyositis, but also rarely occurs in the setting of malignancy-associated dermatomyositis. Calcinosis cutis is also less likely to be found in adult-onset dermatomyositis. In malignancy-associated dermatomyositis, panniculitis can precede, concur, or follow the diagnosis of malignancy. We report the case of a 26-year-old Thai female with calcific panniculitis in the setting of adult-onset dermatomyositis associated with nasopharyngeal cancer. The clinical course of calcific panniculitis in our case was not parallel to the course of malignancy. Calcific panniculitis can appear many years after, despite the remission of the cancer. A thorough review of the previously reported literature is also provided.

## Introduction

Panniculitis is an uncommon cutaneous manifestation in dermatomyositis. Since 1924, fewer than 30 cases of panniculitis-associated dermatomyositis have been reported. It not only occurs in idiopathic dermatomyositis, but also rarely occurs in the setting of malignancy-associated dermatomyositis (Girouard et al. 2012). To our knowledge, only 4 cases of panniculitis in the setting of malignancy-associated dermatomyositis have been documented.

## Case report

**Figure 1:**
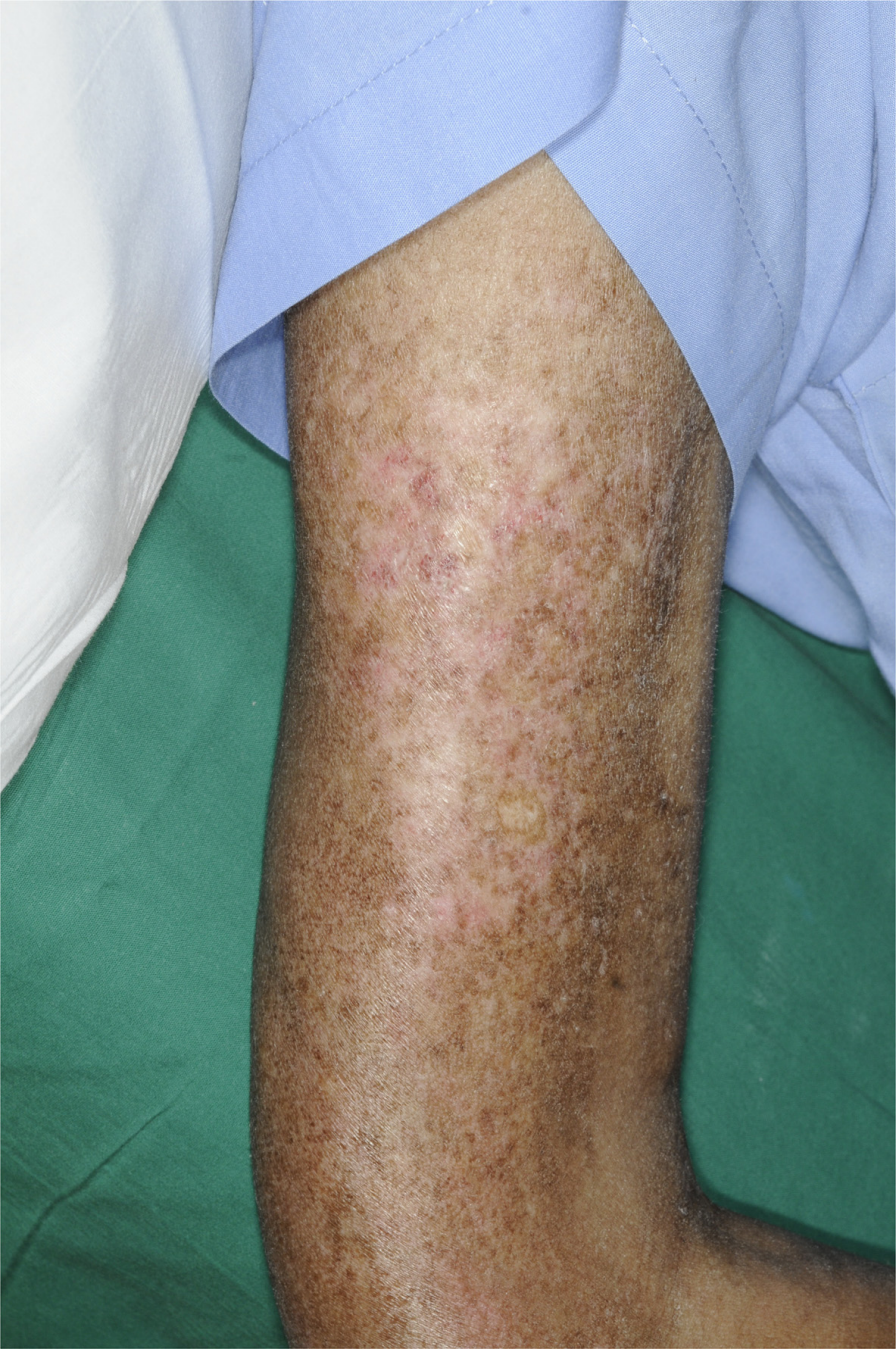
Calcinosis cutis on right arm characterized by multiple, hard to bony consistency, dermal to subcutaneous plaques.

**Figure 2:**
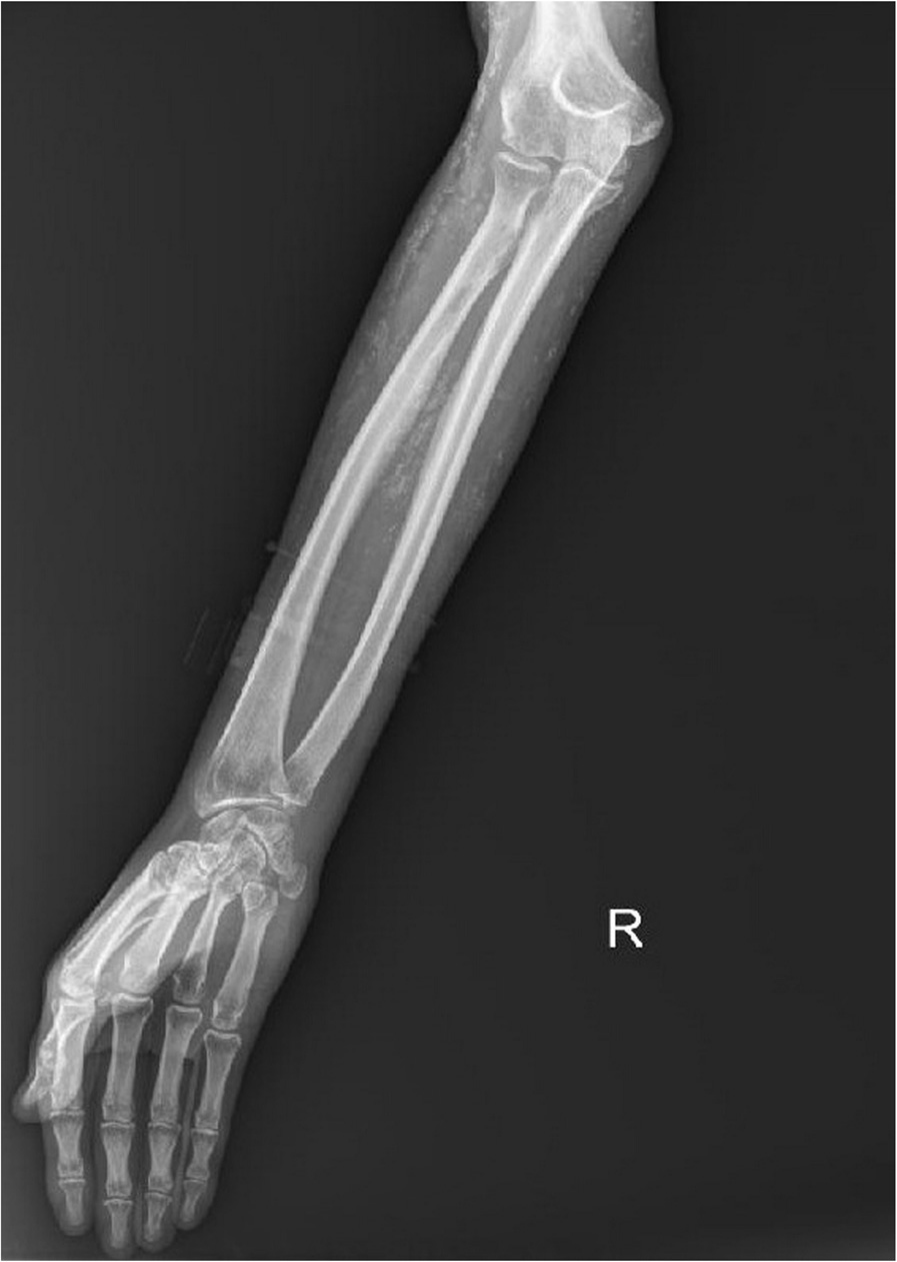
Plain radiographs showing soft-tissue calcification along right arm.

**Figure 3:**
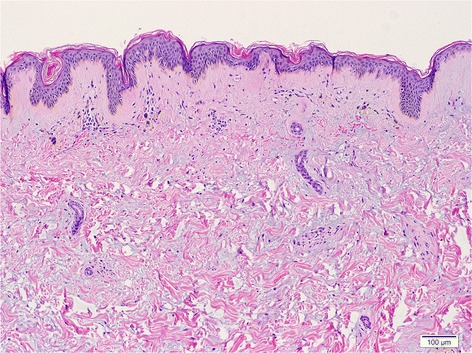
Biopsy showing basal vacuolisation with melanin incontinence and dermal mucin deposition (H&E, original magnification x10).

A 26-year-old Thai female presented with Gottron’s papules, heliotropes, and proximal muscle weakness for 3 months. Investigations showed elevation of creatine phosphokinase, lactic dehydrogenase, and positive antinuclear antibodies (ANA) at the titer of 1:320 (fine speckled pattern). However, negative results were found for anti-dsDNA, anti-Sm, anticardiolipin antibodies, and antiβ_2_ glycoprotein1antibodies. Lupus anticoagulant and complement level (C3, C4) were normal. Myositis-specific and associated antibodies, including anti-Mi2, anti-Ku, anti-PM-Scl-100, anti-PM-Scl-75, anti-Jo-1, anti-PL-7, anti-PL-12, anti-Ro-52, anti-SRP, anti-EJ, and anti-OJ, were all negative. Electromyography (EMG) study demonstrated increased duration of small polyphasic motor unit action potential (MUAP) with early motor unit recruitment, which is compatible with myositis. With 4 of 5 Bohan and Peter diagnostic criteria (Bohan and Peter 1975a, b) for dermatomyositis being met, a diagnosis of dermatomyositis was made without performing muscle biopsy. The patient was treated with chloroquine 250 mg/day, azathioprine 100 mg/day, and prednisolone 30 mg/day. One month after diagnosis of dermatomyositis, our patient was found to have a posterior pharyngeal wall mass and was diagnosed as non-keratinizing nasopharyngeal carcinoma stage IV (T3N3bN0). Following a course of concurrent chemoradiation, nasotelescopy was performed and remission of the malignancy was confirmed. Two years after remission, Gottron’s papules, heliotropes, and muscle power improved, but she developed an ill-defined indurated plaque on her right arm. There was no history of previous trauma to the area. Magnetic resonance imaging (MRI) then revealed diffuse inflammatory process involving skin along right upper arm to proximal forearm, with underneath subcutaneous fat necrosis and marked skin thickening. Five months later, she experienced progressive hardening of skin on her arms, legs, and abdomen. On physical examination, there were multiple, non-tender, fixed, hard-to-bony consistency, dermal to subcutaneous nodules and plaques on axillae, arms (Figure 1), legs, and left lower quadrant of abdominal wall. The lesions varied in size from 1.5 to 5 cm and were mild tender on palpation. Plain radiographs showed soft-tissue calcification along extremities (Figure 2). A biopsy of subcutaneous nodule on her right upper extremity revealed calcification, degeneration of subcutaneous fat cells, and septal fibrosis underneath basal vacuolar degeneration with melanin incontinence and dermal mucin deposition (Figure 3). Lipomembranous change was observed in subcutaneous fat. Serum calcium and phosphate level were normal. Colchicine 0.6 mg/day was initiated for the treatment of calcinosis, without significant change in the lesions.

## Discussion

Adult patients with dermatomyositis are more likely to develop panniculitis than children. Clinical presentation includes painful subcutaneous nodules, indurations, plaques, and/or lipoatrophy. The presence of panniculitis may precede, concur, or occur up to 5 years after diagnosis of dermatomyositis. Table 1 shows characteristics of documented cases of adult-onset dermatomyositis-associated panniculitis. In our case, the patient developed indurated plaques on her arms 2 years after being diagnosed with dermatomyositis.

**Table 1:** **Characteristics of documented cases of adult-onset dermatomyositis-associated panniculiti**s

**Case no./Sex/ Age, y**	**Reference no.**	**Temporal relationship of panniculitis and dermatomyositis**	**Associated malignancy**	**Temporal relationship of panniculitis and malignancy**	**Number and location**	**Autoantibodies**	**Panniculitis features**	**Presence of calcinosis**
1/F/78y	(Lorenzo et al. 1998)	5 months earlier	no	-	N/A	N/A	N/A	N/A
2/F/22y	(Weber and Gray 1924)	Concurrent	N/A	-	N/A	N/A	N/A	N/A
3/F/44y	(Chao and Yang 2000)	2.5 months earlier	NS	-	Multiple/shoulders, back, chest, abdomen, buttock, and bilateral thighs	ANA 1:80 (speckled pattern)	NS	NS
4/F/24y	(Winkelmann et al. 1990)	4 months earlier	no	-	Single/left arm	Negative	Lobular panniculitis with fat necrosis	NS
5/F/42y	(Fusade et al. 1993)	10 months earlier	no	-	Multiple/buttocks, thighs, arms, abdomen, breasts	Negative	Lobular panniculitis with fat necrosis	NS
6/F/23y	(Carneiro et al. 2007)	Later	no	-	Multiple/arms	ANA 1:40 (speckled pattern)	Lobular panniculitis, NS	NS
7/M/19y	(Carrera et al. 2006)	15 months later	no	-	Several/left thigh	Negative	Lobular panniculitis resembling cytophagichistiocytic panniculitis	NS
8/F/40y	(Feldman et al. 1983)	1 year later	NS	-	N/A	N/A	Septal panniculitis	NS
9/F/54y	(Molnar et al. 1998)	Concurrent	no	-	Multiple/arms	Negative	Panniculitis, NS	NS
10/F/57y	(Molnar et al. 1998)	Concurrent	no	-	Multiple/buttocks, left thigh and sacral	ANA 1:32 (nucleolar pattern)	Lobular panniculitis	NS
11/F/60y	(Nakamori et al. 2003)	8 months earlier	no	-	Several/arms	ANA 1:640	Lobular panniculitis	NS
12/F/73y	(Abdul-Wahab et al. 2009)	4 months later	no	-	Multiple/anterior thighs and upper arms	NS	NS	yes
13/F/50y	(Abdul-Wahab et al. 2009)	18 months later	NS	-	Multiple/extensor of all extremities	ANA (speckled pattern)	NS	yes
14/F/29y	(Carneiro et al. 2007)	Concurrent	NS	-	Multiple/thighs	ANA 1:1024 (speckled pattern)	Lobular panniculitis with fat necrosis	yes
15/M/42y	(Lee et al. 1999)	1 year earlier	no	-	Multiple/left buttock and left inguinal area	Negative	Fat necrosis with membranocystic change	yes
16/F/42y	(Solans et al. 2002)	17 months later	no	-	Several/right and left upper elbow	Negative	Lobular necrotizing panniculitis	yes
17/F/80y	(Solans et al. 2002)	10 months later	no	-	Single/right inner elbow	ANA 1:640 (speckled pattern)	Lipomembranous change	yes
18/F/65y	(Ishikawa et al. 1996)	Concurrent	no	-	Multiple/buttocks, left thigh, lower right legs	Negative	Lipomembranous change	yes
19/F/60y	(Carroll et al. 2014)	2 year later	NS	-	Multiple/thighs and buttocks	NS	Lobular fat necrosis with PMN infiltration	yes
20/F/35y	(Lin et al. 2006)	8 months earlier	no	-	Multiple/right arm	Negative	Lipomembranous change	no
21/F/56y	(Lin et al. 2006)	2 year after	Parotid carcinoma	NS	Multiple/arms	ANA 1:640 (speckled pattern)	Lipomembranous change	no
22/M/51y	(Kuriya et al. 1985)	14 months earlier	Rhabdomyosarcoma	N/A	Single/buttock	Negative	Panniculitis with fat necrosis	N/A
23/F/52y	(Leung et al. 2005)	Concurrent	Rectum carcinoma	NS	Multiple/thighs	NS	Lobular panniculitis, NS	NS
24/F/63y	(Girouard et al. 2012)	25 months earlier	Ovarian cancer	Panniculitis developed 18 years after diagnosis of malignancy	Multiple/arms and thighs	ANA 1:160 (speckled pattern)	Lobular panniculitis, NS	NS
25/F/26y	Case report	26 months later	Nasopharyngeal cancer	Panniculitis developed 25 months after diagnosis of malignancy	Multiple/extensor of all extremities, abdomen	ANA 1:320 (speckled pattern)	Lipomembranous change	yes

NS, not specified in report; N/A, data is not available.

ANA, antinuclear antibodies.

Although panniculitis is an uncommon presentation in adult-onset dermatomyositis, microscopic changes in adipose tissue were more common than clinically observed (Chao and Yang 2000; Girouard et al. 2012). Panniculitis was found in up to 7% of skin biopsy specimens from poikilodermatous skin change in dermatomyositis patients (Janis and Winkelmann 1968). Among dermatomyositis cases that had panniculitis, lobular panniculitis with lymphoplasmacytic infiltration, lipomembranous panniculitis, and calcific panniculitis were reported (Girouard et al. 2012; Yamamoto et al. 2007). Consistent with the review by Solans et al. (2002), our case demonstrated degeneration of subcutaneous fat cells, septal fibrosis, and lipomembranous change in subcutaneous tissue underneath basal vacuolar degeneration at the dermoepidermal junction.

In malignancy-associated dermatomyositis, panniculitis can develop from 14 months prior to the diagnosis of malignancy to 4 months after the diagnosis of malignancy (Girouard et al. 2012). Reported associated malignancies include parotid carcinoma, rhabdomyosarcoma, and ovarian adenoma (Girouard et al. 2012). In our case, panniculitis and skin calcinosis occurred 2 years after the diagnosis of nasopharyngeal carcinoma.

In contrast to panniculitis in dermatomyositis, calcinosis cutis is less likely to be found in adult-onset than in juvenile-onset dermatomyositis. Calcinosis cutis presents in up to 20% of adult-onset cases, as compared to 70% of juvenile-onset dermatomyositis cases (Gutierrez and Wetter 2012). Extremities and trunk are the common sites of involvement (Gutierrez and Wetter 2012). Panniculitis is considered the preceding manifestation of the calcific process in dermatomyositis. In our case and consistent with this tendency, there was history of diffuse inflammatory process involving skin along the right arm, with MRI confirming soft tissue inflammation and subcutaneous necrosis prior to the development of calcification. This is consistent with dystrophic calcification. Severity of dystrophic calcification in dermatomyositis ranges from localized small subcutaneous nodules, tumoral deposits, and intramuscular and fascial calcification to severe forms of exoskeleton formation (Gutierrez and Wetter 2012; Reiter Reiter et al. 2011).

## Conclusion

In conclusion, we report the first documented case of calcific panniculitis with lipomembranous change in the setting of adult-onset dermatomyositis associated with nasopharyngeal cancer. The clinical course of our case was not parallel to the course of malignancy. Calcific panniculitis can appear many years after, despite the remission of cancer. However, the association between calcific panniculitis and malignancy-associated dermatomyositis cannot be ascertain by a single case report. Further study and larger case series are needed.

## Consent

Informed consent was obtained from the patient for the publication of this report and any accompanying images.

## References

[CR1] Abdul-Wahab A, Holden CA, Harland C, Patel S (2009). Calcific panniculitis in adult-onset dermatomyositis. Clin Exp Dermatol.

[CR2] Bohan A, Peter JB (1975). Polymyositis and dermatomyositis (first of two parts). N Engl J Med.

[CR3] Bohan A, Peter JB (1975). Polymyositis and dermatomyositis (second of two parts). N Engl J Med.

[CR4] Carneiro S, Alvim G, Resende P, Auxiliadora Jeunon Sousa M, Cuzzi T, Ramos-e-Silva M (2007). Dermatomyositis with panniculitis. Skinmed.

[CR5] Carrera E, Lobrinus JA, Spertini O, Gherardi RK, Kuntzer T (2006). Dermatomyositis, lobar panniculitis and inflammatory myopathy with abundant macrophages. Neuromuscul Disord.

[CR6] Carroll M, Mellick N, Wagner G (2014) Dermatomyositis panniculitis: A case report. Australas J Dermatol. doi:10.1111/ajd.12172 10.1111/ajd.12172 24689899

[CR7] Chao YY, Yang LJ (2000). Dermatomyositis presenting as panniculitis. Int J Dermatol.

[CR8] Feldman D, Hochberg MC, Zizic TM, Stevens MB (1983). Cutaneous vasculitis in adult polymyositis/dermatomyositis. J Rheumatol.

[CR9] Fusade T, Belanyi P, Joly P, Thomine E, Mihout MF, Lauret P (1993). Subcutaneous changes in dermatomyositis. Br J Dermatol.

[CR10] Girouard SD, Velez NF, Penson RT, Massarotti EM, Vleugels RA (2012). Panniculitis associated with dermatomyositis and recurrent ovarian cancer. Arch Dermatol.

[CR11] Gutierrez A, Wetter DA (2012). Calcinosis cutis in autoimmune connective tissue diseases. Dermatol Ther.

[CR12] Ishikawa O, Tamura A, Ryuzaki K, Kurosawa M, Miyachi Y (1996). Membranocystic changes in the panniculitis of dermatomyositis. Br J Dermatol.

[CR13] Janis JF, Winkelmann RK (1968). Histopathology of the skin in dermatomyositis. A histopathologic study of 55 cases. Arch Dermatol.

[CR14] Kuriya N, Kinoshita N, Yokoyama N (1985). Dermatomyositis with rhabdomyosarcoma and panniculitis: report of autopsy case. Nippon Naika Gakkai Zasshi.

[CR15] Lee MW, Lim YS, Choi JH, Sung KJ, Moon KC, Koh JK (1999). Panniculitis showing membranocystic changes in the dermatomyositis. J Dermatol.

[CR16] Leung YY, Choi KW, Ho KM, Kun EW (2005). Disseminated cutaneous infection with Mycobacterium chelonae mimicking panniculitis in a patient with dermatomyositis. Hong Kong Med J.

[CR17] Lin JH, Chu CY, Lin RY (2006). Panniculitis in adult onset dermatomyositis: report of two cases and review of the literature. Dermatol Sinica.

[CR18] Lorenzo JA, Hernandaz J, Baez O, Castro I, Torrado RM, Rodriguez J (1998). Paniculitis lobulillar como forma de presentacion de una dermatomiositis:presentacion de un caso y revision de la literatura. Actas Dermatosifilogr:400-404.

[CR19] Molnar K, Kemeny L, Korom I, Dobozy A (1998). Panniculitis in dermatomyositis: report of two cases. Br J Dermatol.

[CR20] Nakamori A, Yamaguchi Y, Kurimoto I, Kira M, Kosaka H, Itami S, Yoshikawa K (2003). Vesiculobullous dermatomyositis with panniculitis without muscle disease. J Am Acad Dermatol.

[CR21] Reiter N, El-Shabrawi L, Leinweber B, Berghold A, Aberer E (2011). Calcinosis cutis: part I. Diagnostic pathway. J Am Acad Dermatol.

[CR22] Solans R, Cortes J, Selva A, Garcia-Patos V, Jimenez FJ, Pascual C, Bosch J, Vilardell M (2002). Panniculitis: a cutaneous manifestation of dermatomyositis. J Am Acad Dermatol.

[CR23] Weber FP, Gray AMH (1924). Chronic relapsing polydermatomyositis with predominant involvement of the subcutaneous fat. Br J Dermatol.

[CR24] Winkelmann WJ, Billick RC, Srolovitz H (1990). Dermatomyositis presenting as panniculitis. J Am Acad Dermatol.

[CR25] Yamamoto T, Furuhata Y, Tsuboi R (2007). Lipomembranous changes and calcification associated with systemic lupus erythematosus. Clin Exp Dermatol.

